# Evaluation of apparently healthy Egyptian infants and toddlers on the bayley-III scales according to age and sex

**DOI:** 10.1186/s13052-024-01635-8

**Published:** 2024-04-14

**Authors:** Zeinab M. Monir, Ebtissam M. Salah El-Din, Wafaa A. Kandeel, Sara F. Sallam, Eman Elsheikh, Mones M. Abushady, Fawzia Hasseb Allah, Sawsan Tawfik, Dina Abu Zeid

**Affiliations:** 1 Child Health Department/Medical Research and Clinical Studies Institute, National Research Centre, 33 El Bohouth st, 60014618 Cairo, Dokki Egypt; 2 Biological Anthropology Department/Medical Research and Clinical Studies Institute, National Research Centre, 60014618 Cairo, Dokki Egypt; 3 Child Health Department Medical Research and Clinical Studies Institute, National Research Centre, P.O.12622 Cairo, Egypt

**Keywords:** Bayley III, Gender, Age, Cognition, Brain development

## Abstract

**Background:**

Child development is shaped throughout the first years of life through the interaction of genetics and the environment. Bayley-III is valuably used to determine early developmental delay (DD). The aim of this study was to detect the differences in performance of a sample of apparently healthy Egyptian infants and toddlers on the Bayley-III scales in relation to their age and gender.

**Methods:**

This was a cross-sectional study. Bayley scales were applied to 270 of the 300 recruited children following the inclusion criteria; to avoid potential risk factors affecting development. Assessment included cognitive, language and motor skills. Engaged children aged 18–42 months were divided into 4 age groups with six-month intervals.

**Results:**

Approximately 78.4%, 76.2%, and 72% of the participants had average and above average scores in the cognitive, motor, and language domains, respectively. The language domain was characteristically impacted. The oldest age group (36–42 months) scored the highest means composite scores, while the 2nd group aged 24 - <30 months, scored the lowest means in the three evaluated domains. In general, girls had non-significantly higher composite scores than boys, with a small effect size (d = 0.2–0.4). In the language domain, girls aged 30 to < 36 months scored significantly higher composite scores than boys (*p* < 0.05), with a medium effect size (d = 0.73).

**Conclusion:**

The study indicates that the performance of apparently healthy Egyptian children on the Bayley III evaluation differs in relation to age and sex. The most vulnerable age group at potential risk of DD was children aged 24–30 months. Efforts must be directed to investigate the nutritional, physical, psychological and safety needs of this group. Attention must be paid to early childhood intervention programs that stimulate development, especially language development, and they must be tailored on the basis of age and gender. Gender-specific norms may be needed in the evaluation of language development.

## Introduction

The development of children is shaped during the first years of life, which lays the foundations for productivity and well-being later in life. However, this period is also considered to be the most vulnerable period [1]. The quality of the childrearing environment is critical for healthy development. A safe nurturing environment, positive interactions and sharing time with parents are in a positive direct relation to the child’s development [2]. Negative influences such as poverty, poor nutrition, under-stimulating environments and unhealthy surroundings can impede and restrain development [3]. It has been found that the prevalence of childhood delay varies from 10% in Central Asia and Europe to 42% in Central and West Africa [4]. In the USA, developmental delay was found to be 17% more common between the ages of 3 and 17 in boys than in girls [5]. In Egypt, a national community-based study proved that the prevalence of developmental delays in Egyptian children aged 1–12 years is approximately 6.7%, with a higher incidence among boys than girls [6].

During stages of child development, children go through several changes in different domains of physical, communication/language, social/emotional, and intellectual/cognitive development. Particular changes that occur at specific ages of life are called developmental milestones [7].

The lack of information about the common age at which healthy children attain different developmental milestones and whether milestones are attained similarly across sexes remains a principal barrier to defining developmental difficulties and starting intervention efforts in low- and middle-income countries [8].

The developmental assessment of infants is a complicated and time-consuming process. Standardized tools provide a means of evaluating a young child’s development and comparing this to a standardized norm [9]. The Bayley Scales of Infant and Toddler Development 3rd Edition (Bayley-III), with scales for cognition, motor and language development, provides a gold standard developmental assessment tool in clinical and research fields to assess development in infancy and early childhood (0–42 months) [10]. The Bayley III normative population was an American sample of children who were stratified into different age groups, but the test lacked separate gender-specific norms [10].

Some studies have raised concerns about the underestimation of Bayley-III in identifying children with developmental delay compared to Bayley-II [11–13]. However, these studies were mainly conducted in premature infants and specific age ranges. The results from the Bayley scales must be interpreted carefully for all age ranges and in different contexts, taking into consideration the sociodemographic and cultural differences from the standardized American norms.

Neuroscience research has reported sex-based differences in brain structure and function induced by the action of gonadal sex hormones or genes found on sex chromosomes [14]. The National Institute of Mental Health recommended that sex should be incorporated as a significant variable in experimental and clinical studies [15]. The performance of boys and girls may be affected by sex differences in neuronal structures, neurochemistry, neuroanatomy and connectivity [16]. Previous research on sex differences in early childhood development is inconclusive; some studies have revealed gender differences in a number of functions or behaviors [17, 18]. A recent national Egyptian study showed that boys were 1.75 times more likely than girls to be diagnosed with any developmental delays (OR = 1.75, CI: 1.61–1.89) [6], and other research did not find any effect of gender [19]. These differences may be due to variances in methodological issues. Some of the psychological tests other than Bayley-III comprise gender-specific subgroup norms, such as the Language Development Survey [20] and the MacArthur-Bates Communicative Development Inventories [21].

The aim of this study was to detect the differences in performance of a sample of apparently healthy Egyptian infants and toddlers on Bayley scales in relation to age and gender. Accordingly, attention can be directed with more efforts from caregivers, early childhood facilities and governmental institutions toward the most vulnerable age group and gender.

### Subjects and methods

#### Sample size

The sample size was ascertained utilizing epi Info-Statcalc version 7 [22]. The power of this study to calculate the needed sample size was set at 80%, denoting the probability of finding a difference when a difference exists. A sample size of 240 infants and toddlers was estimated. This number was estimated to calculate a true difference of 15% (denoting the probability of finding a difference when no difference existed, i.e., margin of error: ± 0.15) [23]. Then, 10% was added for the expected losses. Determination of the sample size depended on the previously estimated prevalence of developmental delay in different countries, which varied from 10% in Central Asia and Europe to 42% in Central and West Africa [4]. This great variability provided the basis for the sample size calculation, which ensured the largest sample size for detecting any significant difference.

#### Study design and time frame

This was a cross-sectional study in which three hundred children were recruited from September 2017 to September 2019, and Bayley scales were applied to 270 of them following the inclusion and exclusion criteria. Engaged children aged 18 to 42 months were divided into 4 age groups with six-month intervals.

#### Selection criteria

To avoid the effect of potential risk factors affecting child development, Egyptian infants and toddlers were included according to the following exclusion and inclusion criteria. Children with reported prematurity or low birth weight, genetic, congenital, or metabolic disorders, a history of perinatal complications such as intracranial hemorrhage, a history of chronic disease, or severe sensory impairment (auditory or visual) were excluded. Additionally, the child was excluded if he/she was severely malnourished at the time of recruitment (if the height-per age z score (HAZ), weight-per-age z score (WAZ) or body mass index per age z score (BAZ) was less than − 2). Accordingly, infants and toddlers whose gestational age was at least 37 weeks, with a birth weight of at least 2500 g, infants and toddlers who had no physical or mental health issues and subjects who did not use medicine regularly or had chronic disease were included.

#### Ethical issues

The study proposal was approved by the Medical Research Ethical Committee of the National Research Centre, which complies with the International Ethical Guidelines for Biomedical Research Involving Human Subjects [24]. This study was a part of a project of the 11th research plan of the National Research Centre under the title of “A Pilot Study to Evaluate the Performance of Egyptian Infants on the Bayley Scales of Infant and Toddler Development -third edition (Bayley III)” with ID number 11,010,141.

Mothers or caregivers were informed about the purpose of the study, and their permission in the form of written consent was obtained.

Confidentiality: Mothers and children were identified by a serial number, and the information at the individual level was kept strictly confidential.

#### Recruitment of infants was from

- The nursery that belongs to the National Research Centre (NRC).
- Other nearby nurseries.
- The Medical Research Centre of Excellence’s Clinics.

#### Setting

The study was conducted at the Developmental and Behavioral Assessment Clinic at the Medical Research Centre of Excellence, National Research Centre (NRC), Egypt.

#### Measurements

### Background questionnaire

Mothers/caregivers answered a background questionnaire about family sociodemographic data and child characteristics.

### Physical examination and growth assessment

Infants and toddlers were thoroughly examined by expert pediatricians. Growth assessment was performed using anthropometric measurements, including weight (kg), height (cm), and head circumference (cm). The length or height was measured to the nearest 0.5 cm, and the weight was measured to the nearest 0.1 kg using a scale balance with the subject dressed in minimal clothes. Body mass index (BMI) was calculated as weight in kilograms divided by height in meters squared. Head circumference (HC) was measured (cm) around the child’s head to the nearest 0.1 cm. It is the maximum circumference passing around the glabella and the occiput. Each measurement was taken as the mean of three consecutive measurements using standardized equipment and following the recommendation of the International Biological Program [25]. WAZ, HAZ and BMI Z scores were calculated based on the WHO growth standards [26] with the help of the Anthro-Program of PC.

### Assessment of development

Bayley Scales of Infant and Toddler Development (Bayley III), developed by Nancy Bayley in 2006, were utilized to assess the development of infants and toddlers between the age range of 1 month and 42 months [10]. The Bayley scales are described as the most widely used developmental assessment scheme [27]. Bayley-III covers five developmental domains. Cognitive, motor and language tests are administered with the child; interaction, social-emotional and adaptive behavior tests are administered with parent questionnaires. All domain subtests can be administered individually. In the current study, only the cognitive, motor and language domains were assessed directly.

The Cognitive Scale included items that assess sensorimotor development, exploration and manipulation, object relatedness, concept formation, memory, and other aspects of cognitive processing.

The Language Scale is composed of receptive communication and expressive communication items. The receptive communication subtest included items that assess preverbal behaviors, vocabulary development and children’s social referencing and verbal comprehension. The Expressive Communication subtest included items that assess preverbal communication, such as babbling, gesturing, joint referencing, and turn-taking, and vocabulary development, such as naming objects, pictures, and attributes (e.g., color and size).

The Motor Scale is divided into the Fine Motor subtest and the Gross Motor subtest. Fine motor skills included items that measure skills related to visual tracking, reaching, object manipulation, and grasping. The Gross Motor subtest assessed static positioning (e.g., sitting, standing), dynamic movement (e.g., locomotion, coordination), balance, and motor planning.

Scoring for every item is either 1 (credit) or 0 (no credit). Scores available include raw scores, scaled scores, composite scores, percentile ranks and confidence intervals.

The measure with a series of developmental play tasks took between 45 and 60 min to administer. Raw scores of successfully completed items were converted to scaled scores and composite scores. The scores obtained by toddlers were used to determine their performance compared with norms taken from typically developing children. The composite scores are scaled to a metric with a mean of 100, SD of 15 and a range from 40 to 160. The norm-referenced average is from 85 to 115 [10].

#### Statistical analysis

Data analysis was performed using Statistical Package for the Social Science (SPSS) version 21 (SSPS Inc., Pennsylvania, USA). Continuous data are expressed as the mean ± SD, while categorical data are expressed as frequencies and percentages. ANOVA was used to analyse the significant differences between the mean scores of the age groups. The P value was considered statistically significant at *p* < 0.05. Effect sizes were reported as either Cohen’s d or eta squared (η2) for t tests and ANOVAs, respectively. The mean (standard deviation) of the original normative Bayley population was 100 ± 15. Infants were considered below average if a Bayley III score was below 85 on any of the language, cognitive, or motor scales.

## Results

The total number of participants was 270 infants and toddlers. The sociodemographic features of the studied children, including their gender, age and social class, are shown in Table 1. Most of the children were from the middle social class (77.6%), the high social class represented 16.3% and the low social class represented 6.1% of the whole sample.

**Table 1:** Sociodemographic Characteristics of the Studied Participants

Parameter	Number	Percent %
**Sex**		
Male	152	56.3%
Female	118	43.7%
**Age categories**		
from18-<24 months	93	35.2%
from24-<30 months	60	22.7%
from30-<36 months	54	20.5%
from36-42 months	57	21.6%
**Social classes**		
Low social class	17	6.1%
Middle social class	209	77.6%
High social class	44	16.3%

Table 2 illustrates the body mass index of the participants according to the z score; it shows that most of the participants had normal BMI-z scores (92.6%), obese children were approximately 5.3%, and underweight children were only 2.1%.

**Table 2:** Nutritional Status of the Studied Participants According to BMI-Z Score

Parameter	Number	Percent%
**BMI z- score**		
Obese (BMI-z ≥ 2)	14	5.3%
Normal (BMI-z -1.99–1.99)	250	92.6%
Underweight (BMI-z ≤ 2)	6	2.1%

The participants were classified according to the cut-off point of the composite score of the Bayley Scale domains into two groups: one group had average and above average scores (i.e., scores equal to or above 85), while the other group had below average scores (i.e., below 85). We found that approximately 78.4%, 72%, and 76.2% of the participants had average and above-average scores in the cognitive, language and motor domains, respectively (Table 3).

**Table 3:** Classification of Participants into Two Groups According to the Cut-off Point of the Composite Score of Bayley Scales

Developmental domain	Number	Percent%
**Cognitive domain**		
Average and above average	212	78.4%
Below average	58	21.6%
**Language domain**		
Average and above average	194	72.0%
Below average	76	28.0%
**Motor domain**		
Average and above average	206	76.2%
Below average	64	23.8%

### The effect of gender

It is observed that female participants in the whole sample had higher mean composite scores in the three domains than males by 3–4 points, but the differences were statistically insignificant. (94.41 ± 20.56 vs. 91.08 ± 16.53 in the cognitive domain; 93.89 ± 15.97 vs. 90.06 ± 15.15 in the language domain; 97.87 ± 19.77 vs. 93.96 ± 18.06 in the motor domain; *p* > 0.05 in all domains).

Table 4 shows a comparison of the mean composite scores according to gender. Girls had non-significantly higher scores than boys, with a small effect size (d = 0.2–0.4) in all domains in different age ranges, except the age range from 24 to 30 months, where boys scored higher in all domains. Girls had significantly higher scores with a medium effect size (d = 0.73) in the language composite score in the age range from 30 to 36 months.

**Table 4:** Means, standard deviations and effect sizes for the Cognitive, Language and Motor composite scores according to gender

Age categories	Cognitive composite score			Language composite score			Motor composite score		
	Boys M ± SD	Girls M ± SD	Effect size (d)	Boys M ± SD	Girls M ± SD	Effect size (d)	Boys M ± SD	Girls M ± SD	Effect size (d)
18 months to < 24 months	89.29 ± 13.42	91.97 ± 18.40	0.17	90.25 ± 13.79	93.76 ± 15.74	0.24	91.46 ± 11.36	97.83 ± 19.13	0.41
24 months to < 30 months	86.68 ± 13.87	86.05 ± 11.00	0.05	88.27 ± 10.18	86,35 ± 22.75	0.12	87.93 ± 17.22	85.74 ± 17.13	0.13
30 months to < 36 months	92.20 ± 15.59	96.50 ± 27.07	0.19	85.37 ± 16.31	96.37* ± 12.71	0.73	95.84 ± 19.21	102.33 ± 19.81	0.33
36 months to 42 months	98.07 ± 22.38	107.14 ± 14.37	0.44	94.29 ± 19.19	98.07 ± 11.47	0.21	103.30 ± 23.36	105.00 ± 19.87	0.07
Total sample	91.08 ± 16.53	94.41 ± 20.57	0.18	90.06 ± 15.15	93.89 ± 15.97	0.24	93.96 ± 18.06	97.87 ± 19.77	0.21

For an **independent-samples t test**, Cohen’s d is interpreted as follows: d = **0.2** indicates a **small** effect; d = **0.50** indicates a **medium** effect; and d = **0.80** indicates a **large** effect

*p < 0.05 is significant (t test)

**Fig. 1:**
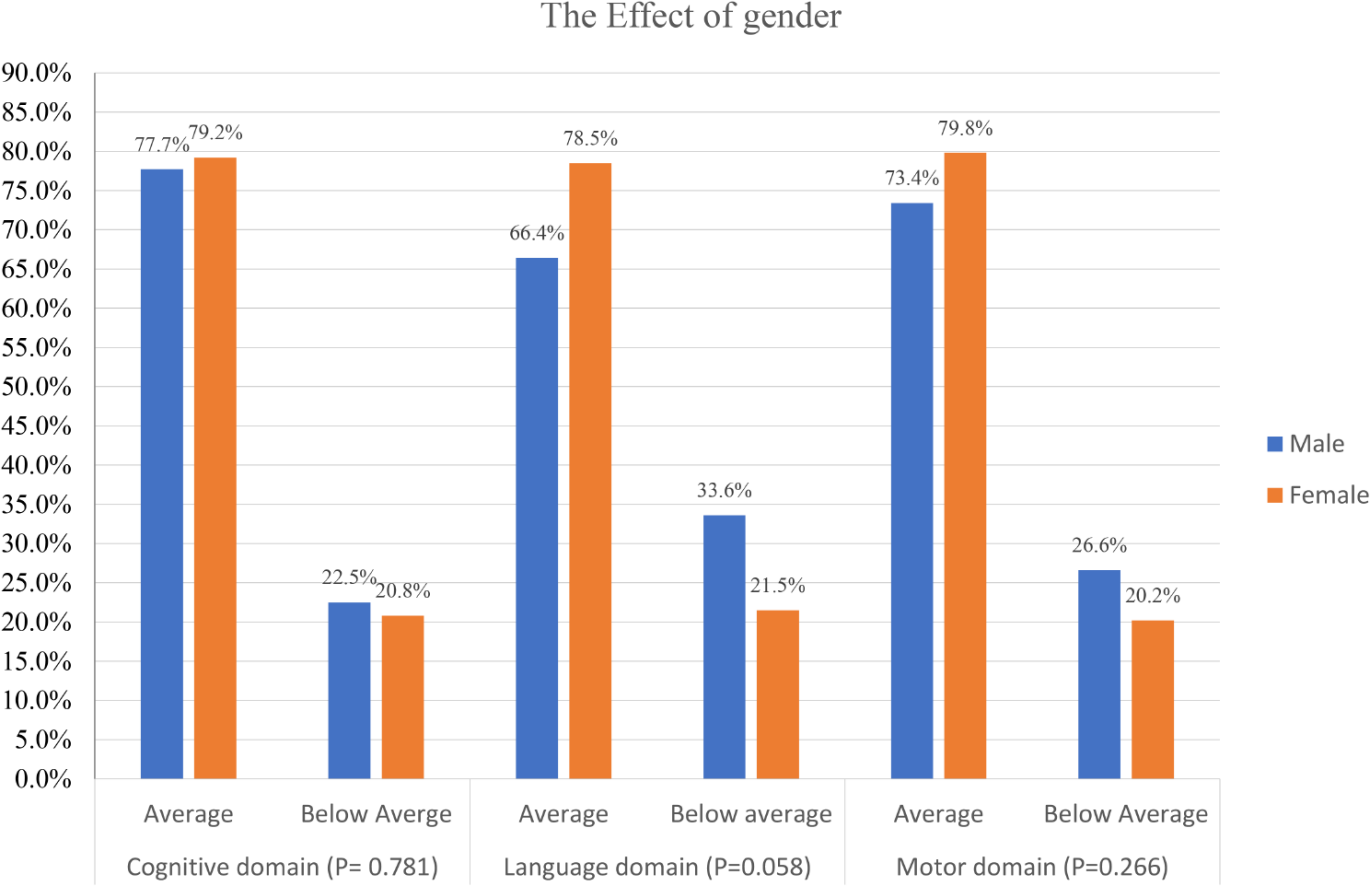
Classification of Male and Female Participants as Having Average and Below Average Scores in The Three Bayley Domains

In addition, on classification of participants according to the cut-off point, the girls had lower percentages of below-average performers than boys, particularly in the language domain, but the difference was not statistically significant (Fig. 1).

### The effect of age

In the present study, participants were classified into 4 age groups, 6 months apart starting from 18 months to 42 months. The mean composite scores of the cognitive, language, and motor domains among the different age categories were compared.

Table 5 shows that the participants in the age range 24 months to < 30 months (group 2) had the lowest composite scores in all domains (cognitive, language and motor). The participants in the age range of 36 months to 42 months (group 4) had the highest composite scores in all domains. There are highly significant differences in cognitive composite scores between different ages.

with medium effect sizes. Additionally, there were highly significant differences in motor composite scores between different age groups with large effect sizes. Meanwhile, the differences in the language composite scores between different age groups were insignificant with small effect sizes.

These results directed attention towards toddlers aged 24–30 months who looked at the potential risk for developmental delay in motor and cognitive development.

**Table 5:** Comparison of mean composite scores of cognitive, language, and motor domains between age categories with effect size

Age categories	Cognitive composite score					Language total composite score					Motor total composite score				
	M ± SD	Std. Error	F	p	η2p	M ± SD	Std. Error	F	p	η2p	M ± SD	Std. Error	F	P	η2p
18 to < 24 months (1)	90.46 ± 15.75	1.69		0.001* 1&4 2&3 2&4	0.07	91.80 ± 14.70	1.58	1.975	0.119	0.03	94.19 ± 15.41	1.68	7.587	0.000* 1&4 2&3 2&4	0.093
24 to < 30 months (2)	86.44 ± 12.74	1.80	5.771			87.57 ± 15.69	2.29				87.08 ± 17.04	2.43			
30 - < 36 months (3)	94.78 ± 23.08	3.26				92.10 ± 15.06	2.15				99.82 ± 19.64	2.81			
36–42 months (4)	101.02 ± 20.40	3.11				95.55 ± 17.49	2.70				103.81 ± 22.14	3.38			
Total sample	92.50 ± 18.50	1.22				91.68 ± 15.64	1.04				95.71 ± 18.93	1.26			

For **ANOVAs** Partial eta squared η2p is interpreted as: η^2^ = **0.01** indicates a **small** effect; η^2^ = **0.06** indicates a **medium** effect; η^2^ = **0.14** indicates a **large** effect

*p < 0.05 is significant

## Discussion

The Bayley-III affords a substantial benefit by providing differential diagnostic information when there are preliminary suspicions of developmental disorders with independent standard scores in the different scales and subtests (cognitive, language, receptive communication, expressive communication, fine motor, gross motor) [28, 29]. In this study, we assessed the performance of apparently healthy Egyptian infants and toddlers on the Bayley-III scales in relation to age and gender.

Within the related literature, there are many national and international studies of various facets of child development using Bayley-III in the evaluation process or as a predictive tool [30–35].

In the current study, it was found that girls had higher scores than boys in all domains, and they had a lower percentage of below-average population. However, the differences were not statistically significant, with a small effect size. In the language domain, girls aged 30 to < 36 months scored significantly higher than boys (*p* < 0.05), with a medium effect size (d = 0.73).

Very few studies have explored gender differences in test scores. Nationally, this study is the first to analyse the gender effect on infants’ performance on the Bayley-III scales. Internationally, many studies have proven the superiority of girls. Wu et al., 2008 [36] reported that female gender was only associated with higher mental and motor scores on Bayley scales. Another study stated that females scored significantly higher on the cognitive, receptive and expressive communication subtests [37]. Moreover, another study found that females scored higher on the fine motor subtest [38]. There are theories that have attempted to explain this gender difference, involving biological and social factors. According to biological theory, sex-based differences in brain structure and physiology reflect gonadal hormone/receptor interactions, their effects within cells, and the intermediating impact of genetic variables, principally the holding of an XX versus an XY genotype [14]. The greater risk of DD in boys is recognized to be linked to hereditary factors in the form of X-linked disorders and the related effects on the central nervous system [39].

Regarding social factors, especially in Asian and African nations, males are still the preferred gender, acquiring more attention and care added to their hasty and aggressive behaviors, making them more liable to diagnosis than females [40, 41]. 

Predomination of girls on boys in language development was observed in the current study, which is on the same track as earlier studies using Bayley-III or other developmental assessment tests. These studies have shown that girls tend to attain a variety of linguistic skills, such as language comprehension, gesturing expression, vocabulary and the capability to combine words, earlier than boys [42–45]. It has been found that parents talk more and in a different way to their daughters than to their sons using a supportive conversation to them, which in turn affords daughters potentially allowing for greater exposure to language [46]. Additionally, one study reported that parental play with girls is unlike that with boys; parents prefer symbolic play with daughters while with sons it is an action-oriented one, which in turn affects both quantity and excellence of language used [47]. In addition, gender distinctions were linked not only to differences in the measured functions but also to differences in the behavior of taking this test between girls and boys. In other words, girls have a better capability to control impulses and attention, while boys are more energetic and active [43, 48].

In this study, we thoroughly investigated the effect of age. It was found that those children in the younger age group (those younger than the age of 30 months) tended to have lower scores than older age groups, with highly significant differences between age groups. The highest composite scores of all subtests and the lowest percentage of below-average children were recorded in the oldest age group (from 36 to 42 months). These outcomes are consistent with Hanlon et al., 2016 [49]. They found that the mean score for each Bayley subscale and the total Bayley Scale were significantly lower in 30-month-old children than in 42-month-old children. The same results were observed by Steenis et al., 2015 [50], but in their study, they related the findings of the scaled scores to the mother’s educational level. They showed that with increasing age, children of mothers with higher levels of education had superior scores on the cognition and receptive communication subtests compared to children of mothers with lower levels of education.

In Egypt, multiple social, economic and nutritional factors could impact the development of young children in several domains. Children aged under 30 months may be constrained within their home environment, lacking the influences of outdoor stimulants. In addition, due to economic causes, Egyptian children may have received fewer opportunities to explore new things, such as toys and picture books, at a young age compared to children in other countries. Most of the mothers in developing countries showed a lack of knowledge on the proper timing of providing different stimulation activities [51, 52]. In addition, a lack of exclusive breastfeeding, as well as malnutrition, can arise, resulting in the risk of differences in social behavior and cognitive and motor development [53, 54]. Gunardi et al., 2019 [55] confirmed that nutritional problems have been linked to developmental delay. A cross-sectional Nigerian study proved a significant association between weight and language and interactive social domains [56]. A meta-analysis study by Sudfeld et al., 2015 [57] found that malnutrition leads to delays in maturation of the auditory system, difficulty in understanding information, apathy and delayed social interaction skills.

Although the majority of participants in the current study had normal physical growth, they still may have had micronutrient deficiency, particularly if they had experienced inappropriate weaning procedures. Deficiencies in iron, zinc, vitamin B12, folate, vitamin A, vitamin D, and iodine can each have severe consequences, including increased susceptibility to infections, reduced growth, cognitive impairment, and decreased school performance in older children [58]. Most micronutrient deficiencies remain undiagnosed due to ambiguous symptoms. However, approximately 56% of preschool children aged 6–59 months worldwide have one or more micronutrient deficiencies [59]. Many national and international studies have confirmed the association between low serum levels of micronutrients and below-average developmental scores [3, 60–62]. 

In the current study, the mean composite score in the language domain was lower than scores in the cognitive or motor domains. It seems that language skills may be particularly impacted in Egyptian and Arab children due to authoritarian parenting styles [50]. Frequent use of commands and criticisms in the early years of life [63], together with a lack of parental responsiveness and support, are associated with delays in children’s language abilities [49–51]. 

Some limitations were noted in this study. The majority of participants belonged to one social class (middle class), so the effect of socioeconomic status on the performance of participants could not be elicited. In addition, there was limited time to study parent‒child relationships and interactions due to the extensive time needed to conduct developmental assessments using the Bayley scales. Studying these variables could have provided an increased understanding of the dynamics and their association with the results. Despite these limitations, the strength of this study was that it was the first study performed in Egypt to discuss the association between age, gender and developmental status to prompt appropriate intervention strategies to support infants and children. This study could be the starting point for more detailed research that would include a larger number of participants, allowing increased generalization.

## Conclusion and recommendations

This study indicates that the performance of apparently healthy Egyptian children on the Bayley III scale differs in relation to age and sex. The most vulnerable age group at potential risk of DD was children aged 24–30 months. Efforts must be directed to investigate and provide nutritional, physical, psychological and safety needs for this group. Proper feeding strategies and dietary diversification for pregnant mothers, infants and young children should be practiced. Fortified foods with an appropriate content of micronutrients should be available, especially to low-income families. Healthcare providers must be sufficiently knowledgeable about child developmental milestones in different age stages and take the initiative to provide precise information to primary caregivers. Attention must be paid to early childhood intervention programs that stimulate development, especially language development, and they must be tailored on the basis of age and gender. Gender-specific norms may be needed in the evaluation of language development.

## Data Availability

The datasets used and analysed during the current study are fully available without restriction upon request from Mones M. Abushady, monesshady@ymail.com.

## Abbreviations

DD: developmental delay
LMICs: Low- and middle-income countries
HAZ: height-per age z score
WAZ: weight-per age z score
BAZ: body mass index per age z score
NRC: National Research Centre
BMI: Body mass index
